# A State-of-Art Review of Digital Technologies for the Next Generation of Tinnitus Therapeutics

**DOI:** 10.3389/fdgth.2021.724370

**Published:** 2021-08-10

**Authors:** Grant D. Searchfield, Philip J. Sanders, Zohreh Doborjeh, Maryam Doborjeh, Roger Boldu, Kevin Sun, Amit Barde

**Affiliations:** ^1^Section of Audiology, The University of Auckland, Auckland, New Zealand; ^2^Eisdell Moore Centre, The University of Auckland, Auckland, New Zealand; ^3^Centre for Brain Research, The University of Auckland, Auckland, New Zealand; ^4^School of Engineering, Computer and Mathematical Sciences, Auckland University of Technology, Auckland, New Zealand; ^5^Augmented Human Laboratory, Auckland Bioengineering Institute, The University of Auckland, Auckland, New Zealand; ^6^Empathic Computing Laboratory, Auckland Bioengineering Institute, The University of Auckland, Auckland, New Zealand

**Keywords:** review, digital, therapy, tinnitus, treatment, technology, biometrics

## Abstract

**Background:** Digital processing has enabled the development of several generations of technology for tinnitus therapy. The first digital generation was comprised of digital Hearing Aids (HAs) and personal digital music players implementing already established sound-based therapies, as well as text based information on the internet. In the second generation Smart-phone applications (apps) alone or in conjunction with HAs resulted in more therapy options for users to select from. The 3rd generation of digital tinnitus technologies began with the emergence of many novel, largely neurophysiologically-inspired, treatment theories that drove development of processing; enabled through HAs, apps, the internet and stand-alone devices. We are now of the cusp of a 4th generation that will incorporate physiological sensors, multiple transducers and AI to personalize therapies.

**Aim:** To review technologies that will enable the next generations of digital therapies for tinnitus.

**Methods:** A “state-of-the-art” review was undertaken to answer the question: what digital technology could be applied to tinnitus therapy in the next 10 years? Google Scholar and PubMed were searched for the 10-year period 2011–2021. The search strategy used the following key words: “tinnitus” and [“HA,” “personalized therapy,” “AI” (and “methods” or “applications”), “Virtual reality,” “Games,” “Sensors” and “Transducers”], and “Hearables.” Snowballing was used to expand the search from the identified papers. The results of the review were cataloged and organized into themes.

**Results:** This paper identified digital technologies and research on the development of smart therapies for tinnitus. AI methods that could have tinnitus applications are identified and discussed. The potential of personalized treatments and the benefits of being able to gather data in ecologically valid settings are outlined.

**Conclusions:** There is a huge scope for the application of digital technology to tinnitus therapy, but the uncertain mechanisms underpinning tinnitus present a challenge and many posited therapeutic approaches may not be successful. Personalized AI modeling based on biometric measures obtained through various sensor types, and assessments of individual psychology and lifestyles should result in the development of smart therapy platforms for tinnitus.

## Introduction

Tinnitus is commonly referred to as “ringing in the ears;” it is the perception of a sound in the absence of a sound source. Tinnitus is the result of a complex cascade of changes within the auditory and emotional networks of the brain that occur following ear or head injury (1). Tinnitus can have mild through to catastrophic effect on life-quality; it can disrupt hearing, attention and sleep, result in anxiety, and depression (2). The incidence of significant tinnitus is highest amongst older populations (3). There is currently no cure for tinnitus, largely due to its heterogeneity (4). Much of the severity of tinnitus relates to the sufferer's psychological response to abnormal auditory and emotional inputs. Stress, anxiety and depression have been shown to occur with tinnitus or contribute to a greater, negative and sustaining reaction to tinnitus (5). Cognitive processes are suspected to affect how dominating tinnitus may or may not become, how easy or difficult it may be to ignore, and whether attention can easily be diverted away from, or is captured by, this endogenous signal (6). In the absence of effective medication for tinnitus a combination of sound-based therapy, to disrupt auditory processing of the tinnitus signal, and counseling therapy to reduce the potentiation of the tinnitus signal by emotional neural networks, has become common (7, 8). This therapy is usually provided by audiologists and is demanding of both clinician and patient time. Its benefits are often only apparent after months and may require the expense of HAs (9). Such sound therapy is very beneficial for some patients but is of limited or no benefit for others (10). This means that considerable cost in health delivery is incurred without any certainty of benefit (8).

Digital processing has enabled the development of several generations of technology for treating tinnitus. Computers have been used for tinnitus assessment since the early 1980s (11) and the internet ushered in online information (and misinformation) becoming readily available. But it was the first commercially successful digital HAs in the mid-1990s that marked the beginning of the first generation of tinnitus treatments based around digital technology (12). The first-generations of digital HAs appeared to be more effective tinnitus therapy tools than their analog predecessors (13). The beginning of the Apple iTunes store in 2001 facilitated the availability and knowledge surrounding downloading of sounds to personal music players (e.g., MP3 players, iPods). MP3 players enabled earlier self-help tinnitus masking strategies developed using the SONY Walkman (14) to be replicated, but with a greater playtime, more listening options and longer battery life. Digitization of sound files and MP3 players has continued to enable the rapid prototyping of sound therapies (15). As of April 2021, a search on the Apple iTunes store identified over 100 digital albums identified as “tinnitus relief.” In the early 2000s a stand-alone tinnitus treatment device, resembling an MP3 player, called the Neuromonics was released based on the concept of systematic acoustic desensitization (16). It implemented a two-stage therapy of noise combined with a music calibrated to have a flat spectrum and weighted to the individuals audiogram profile (17).

We identify the second generation of digital tinnitus devices as offering user-selectable therapy options and, in the case of wearable devices, wireless connectivity. With increases in memory and processing capacity HA manufacturers were able to include tinnitus treatment sounds in many of their models, usually consisting of broadband noise derivatives, some synthesizing surf like sounds (18), others fractal sounds (digital chimes) (19). The emergence of “made for iPhone” HAs saw Smart-phone apps able to connect with HAs via Bluetooth (20). At first Bluetooth power demands required an intermediary device receiving the Bluetooth transmission and retransmitting as a lower drain signal to HAs (21). This inconvenience was addressed when direct connection between phone and HAs become possible. The first universal (Android and Apple) connective HA became available in 2017–2018 (22). As Smartphones became popular their onboard MP3 software negated the need for a separate dedicated MP3 player. The ubiquitous nature of the Smartphone enabled tinnitus apps to be developed by manufacturers and third party developers, these first generation apps consisted primarily of sound libraries that extended the range of sounds available to the listener. Online tinnitus clearinghouses (e.g., www.tinnitus.org.uk) and clinic websites (www.tinnitustunes.com) made a wider range of resources available, including podcasts and sound files to download.

The 3rd generation of digital tinnitus technologies emerged from a plethora of new therapy concepts based on specific, putative, neurophysiological mechanisms (1). Third generation therapies attempt to personalize to a characteristic of the users' tinnitus (7). Digital processing algorithms enabled these new therapies to be implemented through HAs, apps, the internet and stand-alone devices. Sound therapies may be based on tinnitus pitch and use notched sound in an attempt to achieve lateral inhibition (23, 24), patterned tones to create desynchronization (25) or be tinnitus replicas for nocturnal habituation (26). Digital processing has also facilitated multimodal stimulation pairing sound with vagus nerve or trigeminal nerve stimulation. Apps and online counseling services have become more interactive (for example chatbots). Despite technological improvements 3rd generation therapies appear to offer little in population-based benefits above 2nd generation (and possibly 1st generation) approaches (27). Almost without exception tinnitus technology has been made available commercially before clinical trials have shown efficacy. It is possible that some of these 3rd generation devices and therapeutics are very effective, but for whom and when is unclear. Certain subsets of the population may be more responsive to one or other therapies, and this may not be static but change with chronification or dynamically through the day to circadian rhythms. To address this variability, we need to be able to ascertain individual differences that predict the most appropriate therapy, and potentially adjust in real-time to a marker of some tinnitus property that can be modified. Recent AI technologies have presented the opportunity to realize such goals (28). In recent years, AI methods have been implemented in a variety of health settings for the purpose of early diagnosis, developing smart therapy platforms and prediction of response to treatment. The rapid growth of AI-driven technologies has allowed tinnitus researchers to consider AI methods and applications to address open questions in tinnitus data analytics, tinnitus management and accelerating decision-making for choosing the best course of treatment, more specifically, toward development of smart therapy. It is our ambit that such smart tinnitus devices represent the 4th generation of the evolution of tinnitus therapeutic technology.

We are on the cusp of a 4th generation of digital tinnitus therapy that we believe will be defined by the incorporation of physiological sensors, multiple therapy options and AI to personalize therapies. This new generation of tinnitus technology coincides with maturing wearable technology, and in particular Hearables. Hearables are ear level wearable computers or computer interfaces (29, 30). While several start-up companies have come and gone, the release of Hearables by large consumer electronics companies and the inclusion of Hearable features into HAs indicate a pathway for tinnitus technology innovation. As awareness for the heterogeneity of tinnitus continues to grow within the scientific community, research is beginning to move toward precise treatment of tinnitus which is tailored for an individual (31, 32). For tinnitus treatments to be truly individualized, one must understand the physiological and psychological biomarkers of tinnitus and how they influence treatment outcome and selection (7). Progress toward precision, data-driven, treatment of tinnitus requires either large datasets to better understand tinnitus heterogeneity or in-depth repeated measures in individuals in which the technology adapts to, or learns, personal preferences and effective decisions (33). AI can be applied to these datasets (34, 35).

In this state-of-the-art review we will catalog and describe technology that has the potential to deliver real-time customized tinnitus treatment that extends beyond the decision making capability and capacity of clinicians alone. Advancements in mobile computing technology enable ecologically valid technology-based interventions tailored to individual needs. The aim of this review is to identify and discuss potential sensors, transducers and algorithms that may comprise the next generations of digital therapies for tinnitus. To capture information in peer reviewed journals, industry whitepapers and forums, a “state-of-art” review was implemented. State-of-the-art reviews are a specific form of review that focus on current issues and new perspectives, often in areas with a need of further research (36). This is an inclusive form of review, that captures information from a wide variety of sources; it does not exclude material based on a quality criterion in the way systematic reviews do. A trade off in sourcing a wide range of information is the inclusion of some material that be of low evidence quality, for example expert opinion.

## Methods

A state-of-art review (36) was undertaken in March 2021 with cataloging of results in April 2021. The aim of the review was: To review proposed and potential sensors, transducers and algorithms that may comprise the next generations of digital therapies for tinnitus. Google Scholar and PubMed were searched for the 10-year period 2011–2021. The search strategy used the following key words: “tinnitus” and [“Hearing aids,” “personalized therapy,” “Artificial intelligence” (and “methods” or “applications”), “Virtual reality,” “Games,” “Sensors,” and “Transducers”] after an initial search an additional search term “Hearables” was added. The reference lists of these articles were searched for additional pertinent articles including in Gray literature (e.g., public domain consultancy documents, consumer electronics magazines and blogs), older articles were included if they provided context. The authors' knowledge of expert topic areas were used to identify gaps in the search outputs and fill these with appropriate source material. Studies were charted according to technology types and purposes: Hearing aids and cochlear implants, Hearables, Internet-based therapies, Dedicated sound and multimodal therapy devices, Apps, Virtual and Augmented reality, EMA, Sensors, and AI. The articles were cross-references if content lay across categories. A narrative was constructed from the chosen material, based around a pragmatic worldview.

## Results

### Hearing Aids

Hearing Aids (HAs) have been used as tinnitus management tools since at least the late 1940s (37) and this continued with the arrival of fully digital aids in the mid 1990s. HA are used in tinnitus management to reduce accompanying hearing handicap, reduce the levels of attention paid to tinnitus, compensate for deafferentation, and by raising the audibility of environmental sounds so that tinnitus can be masked (38). In the early 2000s the United Kingdom National Health Service modernized their hearing aid program, and digital aids became available to NHS patients. It was found that tinnitus outcomes improved with a shift from analog to digital aids (13). The digital benefit was attributed to greater high frequency amplification (13). New digital processing strategies within 2nd generation tinnitus HA and the emergence of made for iPhone HAs (39) may have resulted in some increment improvements in outcomes, although evidence for this is poor. Sound introduced in this next generation of HAs included synthesized natural sounds, such as ocean surf sounds (18). Streamed nature sounds and Broad Band Noise (BBN) have been found to be equally effective over 6 months (40). Digitally-rendered, fractal sounds resembling musical chimes have also been used (41). Most trials of fractal sounds have shown benefit but have been open label, with large individual variability (19, 42).

A scoping review found HAs were beneficial for tinnitus management (43). A Cochrane review of amplification for tinnitus and hearing loss (44) identified only 1 study (45) meeting their quality criteria which compared digital HA to sound generator (maskers), both groups improved on the Tinnitus Handicap Inventory but there was no statistical difference between groups. A follow-up review (46) included eight studies (with a total of 590 participants). Seven of the studies investigated HAs, four combination HAs and three sound generators. There was insufficient evidence to differentiate between outcomes of the sound therapy options and the level of evidence of an overall benefit was low (46). So, while widely adopted for tinnitus control there is little evidence for 2nd generation hearing aid tinnitus efficiency above 1st generation approaches. The current volume of evidence suggests that hearing aid amplification is an effective way to treat tinnitus, but the research that supports this evidence is not of a high quality (18, 43).

Third generation digital hearing aid tinnitus solutions are translations from body-worn dedicated sound therapy devices based on novel neurophysiological theories (described in a subsequent section). In a case example, applied through HAs, Acoustic Coordinated Reset Neuromodulation, a treatment using patterned tones, seemed feasible (47). Notched noise and music have been used in attempts to inhibit tinnitus (23, 24). Notched amplification takes a similar approach to the use of notched music, but in this case amplification of sound is not applied surrounding tinnitus pitch. There is currently no compelling evidence that novel sound processing adds benefit to conventional amplification for tinnitus (48, 49).

The next generations of HAs are going to apply increasing levels of AI and incorporate biosensors (50). Already HAs are beginning to feature better fidelity (51) and have begun to incorporate sensors such as for fall detection (52) mirroring similar developments in fitness trackers and Hearables described later. As hearing loss often accompanies tinnitus HAs are a logical platform for data-driven wearable tinnitus therapy. Efforts to develop cognitively controlled HAs (53) and the development of ear-based EEG (54) could be extended to tinnitus treatment solutions with real-time adjustment based on AI.

### Cochlear Implants

Cochlear implants are surgical acoustic-electrical transducers that comprise a digital signal processor similar to a hearing aid and a multiple electrode array that is inserted into the cochlear (55). The array provides direct stimulation of the auditory nerve through the tonotopic electrode array when hearing aid amplification would be insufficient to improve hearing. Cochlear implants replace the role of HAs when hearing loss is severe (55). Cochlear implants provide electrical stimulation based on sound patterns, but can be considered a Sound Therapy device because they activate auditory pathways. Interest in cochlear implants for tinnitus therapy began in the 1980s (56), but early experience with implantation for tinnitus showed limited success (57). Cochlear implants are becoming more common therapy options when tinnitus accompanies a severe unilateral hearing loss (58) Tinnitus Sound Therapy strategies developed for HAs, for example apps, are also being trialed with implants (59). A systematic review indicated low-level evidence for the benefits of cochlear implants on tinnitus; they appear to help people who had severe tinnitus prior to the implant, but do carry some risk that the implant surgery may exacerbate or initiate tinnitus (60). As knowledge of tinnitus mechanisms advance there are great opportunities to introduce novel stimulation paradigms through cochlear implants that target tinnitus in addition to, or alternatively, to the focus on speech understanding.

### Hearables

Crum (30) likened the ear to a biological “USB” port, presumably with the brain as the “CPU.” A range of sensors and transducers worn in the ear can measure or affect physiology. These ear level devices have become generically known as “Hearables” (29, 30). A Hearable can be defined as a device that fits in the ear that contains a wireless link (29). We prefer to define the Hearable as an ear level wearable computer or computer interface. Both definitions encompass many HAs as well as ear level Bluetooth headphones and fitness trackers. HAs have been reviewed in a separate section primarily because of their established long history in tinnitus therapy. Although HAs and Hearables appear to be converging technologies they can, at present, be separated by their primary consumers, the music listening, fitness focused, younger public (Hearables) and those persons with greater than mild hearing loss (HAs). It is with this in mind that after setting the initial review criteria it was evident that AI and sensors are being, or could be, incorporated into Hearables for tinnitus therapy, consequently “Hearables” was added as a separate search term. The ambit of the search for this section of the review is to scope the development of Hearable design and their current or potential application to tinnitus.

In the mid 2010s several startup companies emerged in the Hearable space to considerable consumer electronics and media attention. Biometric sensors saw the Bragi Dash emerge as a trend setter amongst these first generation Hearables (61). Some of these companies are no longer operating [e.g., Soundhawk (39); Doppler (62) or have moved from manufacturing to focus on AI development as a 3rd party for other manufacturers Bragi (63)]. The 2016 introduction of Apple Air Pods, for which “Live Listen” allows functioning as basic HAs, signaled an important juncture for access to augmented communication (39) and digital tinnitus therapies. Recent Apple accessibility updates to Air Pods include the availability of background sounds, that could be used for tinnitus masking. The recent purchase of the consumer headphone brand Sennheiser by the hearing aid manufacturer and retailer Sonova is a further indication of market convergence and opportunities for capturing market share across the spectrum of hearing, and potentially tinnitus, needs (64).

Hearables could be used as an alternative to HAs as device for 2nd generation therapy, for example wireless access to sound libraries [e.g., Bose Sleepbuds (65)]. Onboard access to internet of voice (29) may enable virtual counselors beyond simple chatbots. The real promise of this technology is the potential to combine biometrics (e.g., EEG, heart rate, temperature, skin resistance, blood oxygen, and stress hormone levels) with auditory or other sensory stimulation (30). These measurements and response could inform status of mental effort, stress, engagement and attention, direction of vision and physical health (30). The types of sensors that would enable Hearables to be used in 4th generation digital therapies are described in greater depth in a later section of this review. In an opinion piece Crum (30) specifically mentions tinnitus as a health application for Hearables. Bragi began to explore the potential of incorporating tinnitus treatments in future generations of their Hearable (66) before moving away from manufacturing. At the time of writing Nuheara was one of the few Hearable companies highlighting hearing loss (67), and had identified tinnitus as a market (https://www.nuheara.com/how-it-helps/tinnitus-relief/).

### Internet-Based Therapies

Internet-based therapies include Internet Cognitive Behavioral Therapy (iCBT), online counseling, peer support and tailored sound. The 1st generation internet was used for tinnitus in a manner similar to a written self-help book, as a repository for information. ICBT programs developed greater content as a form of 2nd generation digital tinnitus treatment. ICBT typically consists of text-based modules for tinnitus patients to work through. An example is the Tinnitus E-program, a 10–12 week self-directed approach consisting of education and information about tinnitus, management, resources available, training for psychological strategies, social support, and monitoring of tinnitus (68). In addition to written information, behavior change techniques such as relaxation methods are available as downloadable MP3 files (68). ICBT in various forms has a good level of evidence to support its use (69). Tinnitus Tunes (www.tinnitustunes.com; established in 2016) is another 2nd generation internet-based digital tinnitus therapy. Subscribers undertake self-directed activities to complement their clinician's advice. It offers a 12-week structured program consisting five separate steps: (1) Education and information so that members can remove any false beliefs they have about tinnitus. (2) Information about role of different clinicians. (3) Managing stress associated with tinnitus (e.g., relaxation sound files, audio podcasts on visualization and progressive relaxation). (4) Training the patient's brain to ignore tinnitus through attention refocusing and adaptation. (5) Prevention of relapse through lifestyle tips. Weekly emails are sent to users that include case studies and lifestyle tips to suit user needs.

Notched sound/music is a 3rd generation online therapy; based on a specific tinnitus mechanism and customized to the individual. Notched sound involves customizing sound (usually music) by removing of sound energy in a band around the patient's reported tinnitus pitch. Two examples are https://www.audionotch.com and https://www.tinnitracks.com/en. The concept is based on experimental work that notched sound may result in lateral inhibition (23). The user chooses the audio signal they wish to have notched (e.g., music or white noise) an online processing algorithm applies the notch, then sounds are played over the computer or downloaded to be played on a portable device. The use of notched sound from the internet or app has only a low level evidence base (70). A double-blinded controlled trial of notched sound found no significant change in the primary outcome of the Tinnitus Questionnaire, although there was a change in a tinnitus loudness rating (24).

### Ecological Momentary Assessments and Mobile Crowdsensing

EMA and mobile crowdsensing have been gathering momentum in tinnitus research in recent years [see recent reviews (71, 72)]. EMA describes the collection of data from participants as they go about their everyday lives, outside of research laboratories or clinical appointments. The ubiquity of smartphones in modern society has made implementation of this approach much more feasible and cost-effective than in the past. Smartphones have in-built sensors, processing, and data transfer capabilities. Participants can be notified via their phone at any time to complete surveys (also delivered on their phone) or data can be collected from the in-built sensors and connected devices such as smart watches (73). The EMA approach stands in stark contrast to the highly controlled environments used in traditional clinical research. These two styles of data collection each have their own strengths and weaknesses, and can serve to inform and compliment the other. With a focus on group effects, traditional clinical research aims to remove as much variability as possible from the testing environment between appointments (within and between participants) in order to try and reduce confounding factors (74). However, these research settings lack ecological validity as they tend not to reflect the participants' everyday environment. EMAs offer ecologically valid measurements at the expense of control over the environment. Interaction between effects and different environments is often an interesting and relevant avenue for research (72, 75). Furthermore, while group studies are undeniably useful for assessing treatments, their methods inherently lack the nuance required for understanding individual differences in conditions and responses to treatments. The EMA approach has been useful in fields investigating conditions of high heterogeneity of symptoms and treatment responses such as psychology and psychiatry (76). One of the reasons that successful tinnitus treatments have remained elusive and of variable benefit is because of the highly heterogeneous nature of the condition in terms of etiology, experience, reaction, and response to treatment (4).

Smartphone-delivered EMA tinnitus studies have shown high engagement from participants, especially from people with more severe symptoms (77–81). A large, longitudinal study (81) found that there was a high drop-off rate after the first few days of initial engagement, but that predictors of continued engagement were evident at these early stages. They suggested that personalized motivators should be considered to increase adherence. This suggests that people will engage with tinnitus-focused EMAs but future apps could increase adherence through more engaging and interactive content, such as gamification of data collection methods (82). The incorporation of sensor data in such apps could lead to more targeted timing of EMAs and perhaps less reliance on subjective survey data (though this data is still highly relevant in tinnitus research at this stage). Software is already being developed to support integration of sensor data with treatment plans and allow information flow between patients, clinicians, and researchers (83).

EMA studies have revealed various factors accounting for within- and between-subject differences in tinnitus perception, including time of day, emotional state and arousal, stress level, concentration, and environmental sound (71, 84, 85). Measures of tinnitus impact (such as annoyance and perceived loudness) can fluctuate over a day, and between days (85). EMAs collected over long periods could be used to examine and identify patterns within patients and potentially enable the development of precision treatments in terms of the timing and targets of treatment.

Data collected from the combination of sensor and EMA technologies has the potential to advance individualized tinnitus treatment and research for patients, clinicians and researchers. Patients could be given more control over their tinnitus by being informed about situations and environments that are likely to increase or decrease their tinnitus symptoms (71). This information could be delivered and interventions suggested in real-time if AI algorithms were developed to predict these situations accurately and produce feedback based on case history, EMA and biosensor data (86). Researchers could also use biosensor data to automate the delivery of EMAs to gain subjective data during high or low stress situations (72). There is potential for data mining opportunities with large anonymized EMA datasets that could detect patterns in data to elucidate tinnitus subtypes, environmental impacts and circadian patterns, and inform treatment.

Mobile crowdsensing and EMA tend to be more cost-effective to implement than clinical trials, and require less labor (especially with increasing automation capabilities likely in the future), have the added benefits of ecological validity, the ability to examine heterogeneous populations, and the elimination of recall bias (84). It has also been suggested that these methods could inform clinical trials. Selection of participants (and therefore sensitivity) could be improved with machine learning used to identify participants most likely to respond to therapies (78). Time of day has been identified as a key consideration for when measurements are taken (84, 85). Accounting for this will improve the accuracy of trial results. EMA and mobile crowdsensing tinnitus research supports the development of interventions that reduce stress and improve emotional stability to improve tinnitus symptoms (71). The research has also revealed the need to further investigate circadian factors in tinnitus, and the underlying chronobiological mechanisms (85, 87).

**Figure 1:**
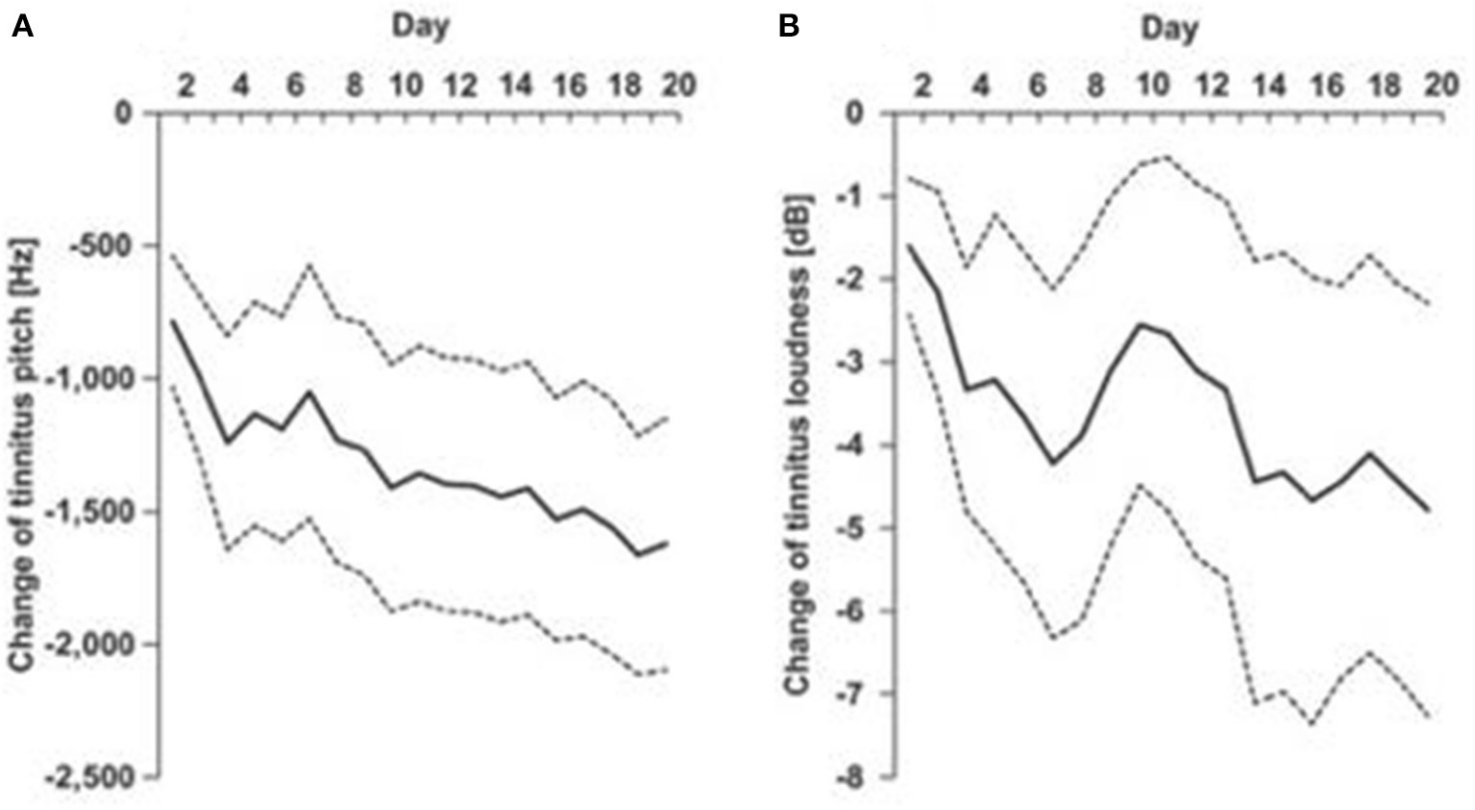
EMA of tinnitus pitch **(A)** and loudness **(B)** recorded each day of a 20 day multisensory training program [Spiegel et al. (89), used with permission of authors].

EMA has been employed in tinnitus using: Palm pilots (77) text messaging linked to surveys (72) a TrackYourTinnitus app on Smartphones (85) and Smartwatches (73), and as part of a serious games (82, 88) and multisensory Training (89, 90) (Figure 1). All these methods for EMA are feasible with response rates to text reminder EMA for tinnitus being high (79–88%) (72, 78). Average time to complete questionnaires on smartwatch were 7.8% longer than on a smartphone (215.4 s & 198.6 s respectively) (85).

Common outcomes among studies include: tinnitus fluctuates within and between days, there is a link between emotional dynamics, stress and tinnitus loudness and distress, and some correlation between increased severity of tinnitus and different times of the day (71) although not all studies showed such variation (77). Tinnitus loudness and distress have been found to be most severe at night and early morning, while stress was most severe in the afternoon (85). The ability to track tinnitus changes over a day may enable individual tailoring in timing of therapeutic interventions, possibly in tune with circadian rhythms (85). In addition to question-based EMA tracking treatment progress through pitch and loudness matching is achievable (82, 88). The addition of biosensors would increase the information being obtained, and with AI, enable real-time adjustments.

### Biosensors

Physiological assessment of function has been part of medical diagnosis for decades. Measures such as blood pressure (BP), heart beat rate (HR), electrocardiograms (ECG/EKG), functional magnetic resonance imaging (MRI) magnetoencephalograms (MEG), and electroencephalograms (EEG) are routinely used in clinics, hospitals and research labs to assess cardiac (BP, ECG), and brain (EEG/MRI/MEG) function. BP, ECG, and EEG along with many other objective physiological, or psychological, function tests can be undertaken in the field, in ecologically valid settings (91, 92). Although there is no single physiological objective measure of tinnitus itself, years of lab based search has identified many related markers of tinnitus related activity, for example neural networks associated with tinnitus (93), measures of emotion, and stress (94). New miniaturized wearable technology is now available to make longitudinal measures of physiological function (95–104) that can be related to behavioral indices of tinnitus.

A strong link between tinnitus and stress has been found epidemiologically, and is seen in clinical practice; patients often report tinnitus onset or increased tinnitus severity in response to stressful events (94). People with tinnitus display dysfunction in both the short-term stress response mediated by the sympathetic nervous system, and the long-term stress response mediated by the hypothalamic pituitary adrenal axis (105). Imaging studies have found increased activity in autonomic brain regions in those with tinnitus, which may act to generate and maintain the tinnitus percept and the related emotional distress (106). If tinnitus related distress is maintained long-term, the sympathetic response may become blunted (105). It is feasible to continuously monitor various physiological variables, including the inter-beat interval (from which heart rate and heart rate variability can be derived), blood volume pulse and skin conductance and relate these to tinnitus (107). Skin conductivity can be used to measure stress, it may be possible to measure cortisol and volatile organic components from the skin (103) possibly using flexible electronic patches (99, 101).

EEG is a non-invasive electrophysiological measure of brain function with high time resolution. In laboratories it is normally recorded from multiple wired electrodes across the skull, often held in position by a cap and electrical conductance enhanced with a conducting gel (97). We are now able to move measures of EEG outside of the lab and record frequently across hours or days, this information can be stored and or relayed back to the researchers. A key feature of wearable EEG is the integration of the electrodes and connecting leads into headsets or ear-level devices (102, 104). Various forms of wearable EEG have been developed and trialed, silver electrodes in a custom earmold (108) cloth electrodes with non-custom earbud (96, 109) conductive silicone electrodes in the ear and around the ear (104) and fingered electrodes to improve contact (97). At present there is a tradeoff between the convenience and usability of these devices and resolution and/or range of applications. Fewer electrodes means brain regions removed from the recording site contribute less to the recorded signal and there is less data. The form of the EEG headset should match the most important spatial region(s) generating the activity of interest, otherwise spatial smearing of activity various brain regions may occur.

Disruption of sleep is a common complaint amongst tinnitus sufferers. Polysomnography (PSG) is the gold standard for objective sleep monitoring (110). PSG uses EEG, electromyography, electro-oculography, electrocardiography, pulse oximetry, and numerous other measures. PSG is undertaken in a laboratory and is expensive and labor intensive. Actigraphy is less expensive method, that can be used at home, and estimates periods of wakefulness and sleep from timing, intensity, and duration of movements using inertial sensors (98). Incorporation of heart rate and variability, skin conductance and temperature along with movement should, depending on algorithms used, improve accuracy (98).

An example of the use of sensors in assessing therapy outcome was a small 12 participant randomized crossover study comparing visualization therapy with visualization paired with self-selected nature sounds. Sleep was assessed using wrist-based actigraphy (Actiwatch 2®) (111). Sleep quality was measured by actigraphy estimates of total sleep time, sleep onset latency, sleep fragmentation, and wake after sleep onset. Sleep onset latency significantly improved following both treatment conditions (111).

Medical monitoring and consumer electronics have converged with the development of smart fitness tracking watches. Much like the convergence of HAs and Hearables this blending of wearable technology is a potent catalyst to apply to the Tinnitus field. Consumer fitness and activity Smartwatches are popular, and provide increasingly accurate measures of function (98). Biosensors, alongside smartphone acquired context such as geospatial information (112), weather conditions and EMA may cue real-time treatment modification (100). Monitoring emotion may enable identification in changes in mood that may necessitate different therapy (96), sensors may be used to provide personalized coaching for health behaviors (95).

### Apps

Several recent reviews have previously identified tinnitus-related apps, although scope, inclusion/exclusion criteria, and definitions differed between them (83, 113–118). Tinnitus patients have been surveyed about their preferences in apps (113), but aside from a small trials (119) evidence of benefit from apps for tinnitus is absent. Sereda et al. (113) found the five most commonly used apps in descending order from highest were: (1) White Noise Free, (2) Oticon Tinnitus Sound, (3) Relax Melodies–Sleep Sounds, (4) myNoise, and (5) Tinnitus Therapy Lite. Due to the fast development and release of new apps on various platforms, the list of apps is ever-growing and changing. Therefore, the number of tinnitus-related apps available at any time is difficult to report but recent reviews have suggested over 200 (116, 118). However, a common observation was that very few apps had been scientifically validated or tested for efficacy, and there was a high risk of bias in many of the studies that were available (83, 117). Furthermore, Sereda et al. (113) found that most of the apps that they reviewed were self-help apps that did involve clinicians. App services identified included CBT, assessments and self-measurement of hearing and tinnitus symptoms, EMA, serious games, and sound therapies (115). App-based tinnitus sound therapies take the form of masking, notched music, and hearing aid control. While these therapies are well-established, there is no guarantee that a given app is implementing them correctly. Similarly, without expert guidance app users may not use the therapies correctly or as intended, and some therapies may not be appropriate for some individuals.

Mobile delivery of therapies has several advantages over traditional clinic-based therapies. Barriers to accessing care such as distance or hesitancy to engage in face-to-face therapy can be overcome. Therapy, questionnaires, and compliance measures can all be implemented on the same device (120). Mobile therapy programs are also generally cheaper to implement, can reach a wider audience, and require less clinician time. Hauptmann et al. (120) found that tinnitus pitch matches measured in clinic vs. in an app did not differ, but the app measures showed less variability than the clinical measures. This is an example of how technology can save time for clinicians and even produce more reliable results. However, clinician involvement is still desirable in order to direct patients to valid and appropriate apps, and instruct them on correct use of therapies (116, 118). Smart technology opens up the possibility of collecting objective data regarding the user's physiology and environment in real-life settings. This can be achieved through the in-built sensors present in modern smartphones (e.g., GPS, microphone, camera, gyroscopes, and accelerometers) (115) as well as peripheral wearable devices such as smart-watches and hearables. However, limitations of hardware and differences between devices must be considered when interpreting the data (121). Other limitations to mobile therapy can include lack of validation, lack of expert supervision, and incorrect use. In research, samples can differ between platforms; newer platforms tend to attract younger participants than traditional advertisements through clinics (122). Drop off in usage after initial sign up is common in internet and mobile based therapy and research (119), although the reasons why are not well-understood (123). Inconsistencies in terminology have been identified as a problem in internet/digital psychological interventions that can lead to miscommunication and can hinder systematic reviews and research (124). Mitigation of inconsistent terminology should be considered in the tinnitus field to enable evidence-based validation of digital therapies. One review even suggested an app review board, similar to journal editorial boards to develop and ensure standards are met before apps can be recommended by clinicians (114).

### Auditory Training and Serious Games

Auditory training is a learning method in which listeners are taught to make perceptual distinctions about sounds being presented. A review undertaken at the beginning of our decade of interest found improvement in outcome measures in nine of ten studies after auditory training, but with low-moderate levels of evidence (125). Frequency discrimination tasks have been the primary training mode (125, 126), but frequency categorization training has been suggested as an alternative method (127). The training tasks may have had benefit through attention mechanisms as opposed to pure sensory improvements in discrimination (6, 125, 128).

Gamification of training may be an important consideration to maintain motivation and compliance with auditory training (128). “Serious games” do not have entertainment as their primary goal but, instead, are intended to change behavior or teach new skills while being engaging and enjoyable (129, 130). A systematic review of application to psychotherapy and meta-analysis of serious games for mental health have indicated, with some caveats to research quality, that serious games are effective (129, 130). A game based on ignoring distractor sounds resembling tinnitus (on that day) while receiving points reward in identifying non-tinnitus target sounds has been developed and tested (82, 88). Users advanced through different levels of an increasing number of distractors across 20 days of 30 min game-play. A feasibility study demonstrated the tasks were achievable and the system was useable; preliminary data indicated significant reduction in tinnitus handicap (88). A controlled trial demonstrated Tinnitus Functional Index scores improved as did performances on audio and visual attention tasks (82). The N1 auditory evoked potential latency was also reduced for sounds remote from tinnitus pitch (82). The concepts of training to focus on target sounds while suppressing background sounds used by Wise and colleagues (6, 82, 88) have been mirrored by other developers (131).

### Dedicated Sound Therapy Devices

Dedicated sound therapy devices are desktop, hand-held or body worn tinnitus devices that have a single purpose: tinnitus treatment. Neuromonics® therapy is a habituation-based, 1st generation passive music sound therapy. The Neuromonics® “Oasis” became available in the mid-2000s as a hand held digital sound player with Bose headphones. It is now available as a download for Smartphones, the “Oasis Pro” (https://neuromonics.com). It uses music that has been spectrally flattened (to reduce bass dominance) and adjusted to the individuals hearing (usually a treble increase). It consists of two stages: stage 1 is noise with modified music, stage 2 is modified music alone. Multiple trials of the treatment have been undertaken indicating success in reducing negative psychological aspects of tinnitus (17, 132). Neuromonics® treatment outcomes have been found to be equivalent to ear-level maskers (133).

Acoustic CR® (Coordinated Reset) Neuromodulation was developed from an electrical stimulation paradigm to treat Parkinson's disease (134). It is available as a handheld processor and wired air conduction headphones: the Desyncra™. The tinnitus treatment consists of temporally patterned tones of frequencies that span a tonal pitch match. The treatment aims to desynchronize aberrant neural ensembles. We consider the Desyncra a 3rd generation treatment, being based on a plausible neurophysiological mechanism and with personalization (tinnitus pitch); however evidence for its benefits are limited (135). An unpublished controlled trial suggested no benefit of the Desyncra treatment over an active control (134). Another 3rd generation device was the Otoharmonics® Levo System. This consisted of tinnitus synthesis software Apple iPod and headphones. It used a tinnitus replica sound that was played during sleep and was based on the hypothesis that tinnitus emerges to replace an input deficit, matched sound should interrupt or reverse this (136). There is limited clinical evidence available demonstrating its efficacy, one trial showed clinically meaningful change in a questionnaire after 3 months (26); the Otoharmonics company appears to have ceased operating.

### Multimodal Therapies

As the name suggests, therapies using multiple modes don't just use sound, but couple it with some other sensory stimulation or nerve modulation. Evidence of multimodal stimulation benefit comes from animal models of tinnitus (137, 138). The MicroTransponder, Serenity® pairs sounds with Vagal Nerve stimulation as an implanted device (139), The Neuromod Lenire couples sound stimulation with tongue tip^TM^ trigeminal stimulation (140). The Neosensory “Duo” combines sound with wrist haptic stimulation (141). These three systems are available clinically in some countries. Computer-based perceptual training has also trialed combining sound, tactile and visual stimuli (89, 90). Clinical outcomes appear variable with questions as to what combination of sound and other stimulation is optimal (142). Further evidence is need to confirm that these treatments offer clinically meaningful benefits above auditory stimulation alone.

### Virtual and Augmented Reality for Tinnitus Therapy

**Figure 2:**
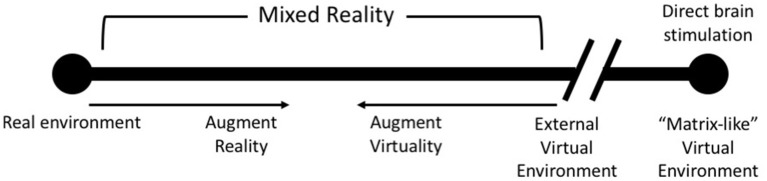
An update of Milgram's Reality-Virtuality Continuum (150) proposed by Skarbez et al. (151) [image redrawn and modified from Skarbez et al. (151), Figure 2, page 3].

AR and VR have been used for the purpose of entertainment (143, 144), enterprise (145) and health care (146, 147). Another popular use for both these forms of technology has been for collaboration in virtual spaces (148, 149). The last decade has seen the emergence of Augmented Reality (AR) and Virtual Reality (VR) as healthcare tools. These emerging technologies are capable of generating immersive environments that leverage the body's perceptual capabilities. While VR provides a completely computer generated environment which isolates a user from the real world, AR incorporates the real world by augmenting the environment, visually and aurally, the user inhabits (150). VR and AR lie on opposite spectrums of the Reality-Virtuality continuum described by Milgram et al. (150) (Figure 2).

Researchers have suggested the use of VR for tinnitus; stating that the ability to expose patients to ecologically valid environments in a safe and regulated manner may help deliver more effective therapies for the treatment of tinnitus (152). Furthermore, at least one study demonstrates the use of VR for the treatment of tinnitus (153). While the results from that study show no differences between Cognitive Behavioral Therapy and VR (153), there are many parameters to explore, particularly the association of simultaneously occurring sensory cues. Most of the technological focus in virtual and augmented reality has been on visual stimuli. But virtual auditory scenes are important for immersive environments. Efforts have been made to create realistic auditory avatars of tinnitus (154) and HRTFs used for spatial rendering of sound to manipulate tinnitus perception through training (82) or masking (155). There is the possibility that by harnessing such auditory signal processing, coupled to vision and haptics, the perceived reality of tinnitus might be changed from an unreal phantom sound in the head to something resembling an ecological valid sound (156).

**Figure 3:**
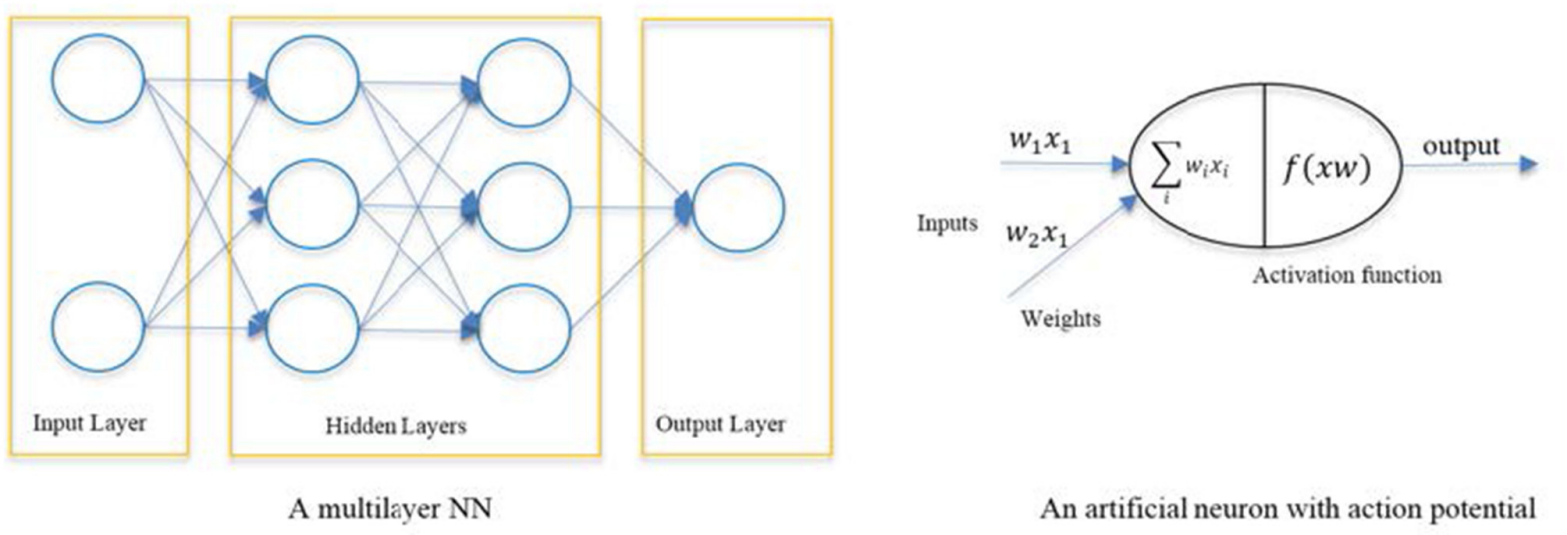
Block diagram showing components of an artificial neuron.

Familiarity with one's surroundings and other factors can significantly affect the efficacy of the treatment that is provided (75). Another important factor to consider is which of AR and/or VR technology is best for the purpose. Both AR and VR have their advantages and disadvantages. For example, with AR we can use the patient's natural surroundings in combination with computer generated auditory and visual cues to deliver therapy. While such an approach offers the best ecological validity, it does not afford complete control of the environment. In cases where control of the virtual environment is desirable in order to manipulate delivery of the therapy, the inherent flexibility of VR can help achieve this with relative ease. More work needs to be done, both with AR and VR, to explore how these technologies can be used in an effective manner for tinnitus. Some of this work could possibly involve exploring how “Matrix-like” direct brain stimulation (Figure 3) could be used to manipulate the interoceptive sense to deliver effective treatment (151).

### Artificial Intelligence: Methods and Applications in Tinnitus

The 4th generation of digital tinnitus therapies will almost certainly require AI to automate functions and make use of multimodal sets of data acquired through biosensors. Developments of new algorithms in AI, and its adoption by healthcare providers, have been transforming the tinnitus field in many ways, with impacts on areas including personalized diagnosis, prognosis and smart therapeutics (157). This section focuses on the state-of-the-art methodological developments in AI in tinnitus studies.

#### Analytical AI Methods for Tinnitus

AI can be used to develop intelligent systems and devices. Smart algorithms can learn from multimodal sets of data to extract meaningful patterns that can indicate certain health outcome (diagnosis and prognosis), more accurately and faster than traditional approaches (157). The application of AI techniques to tinnitus started relatively recently and is so far limited. Thus far, AI algorithms have mostly been used in operational aspects of tinnitus such as comparative analysis (tinnitus vs. control), evaluating tinnitus-related distress, and individualizing tinnitus treatments through feature selection, classification, and prediction tasks (34, 83, 158).

AI has been applied to develop advanced systems (machines) that can learn from data, so-called Machine Learning (ML) in which a variety of computational architectures and learning algorithms have been emerged to increase the accuracy of decision making and decision support (159). The most commonly developed ML systems are based on Artificial Neural Networks (ANN) which loosely model the information processing mechanism observed in neurons in a biological brain (160). ANNs are organized in three main layers of neurons (input layer, hidden layer, and output) (Figure 3). The input layer nodes pass the input data (e.g., biosensors) to the ANN, the hidden layer neurons are computational units that learn from the input data while applying non-linear functions which link the input samples to the proper output neurons (diagnosis labels) (160, 161). State-of-the art in ANN is Deep Learning (DL) in which several hidden layers of neurons are used, each performing automatic extraction informative features from the input data and then pass them to the next layer (162). This is a modern variation of ANN that permits practical application and optimized implementation of non-linear classifications.

**Figure 4:**
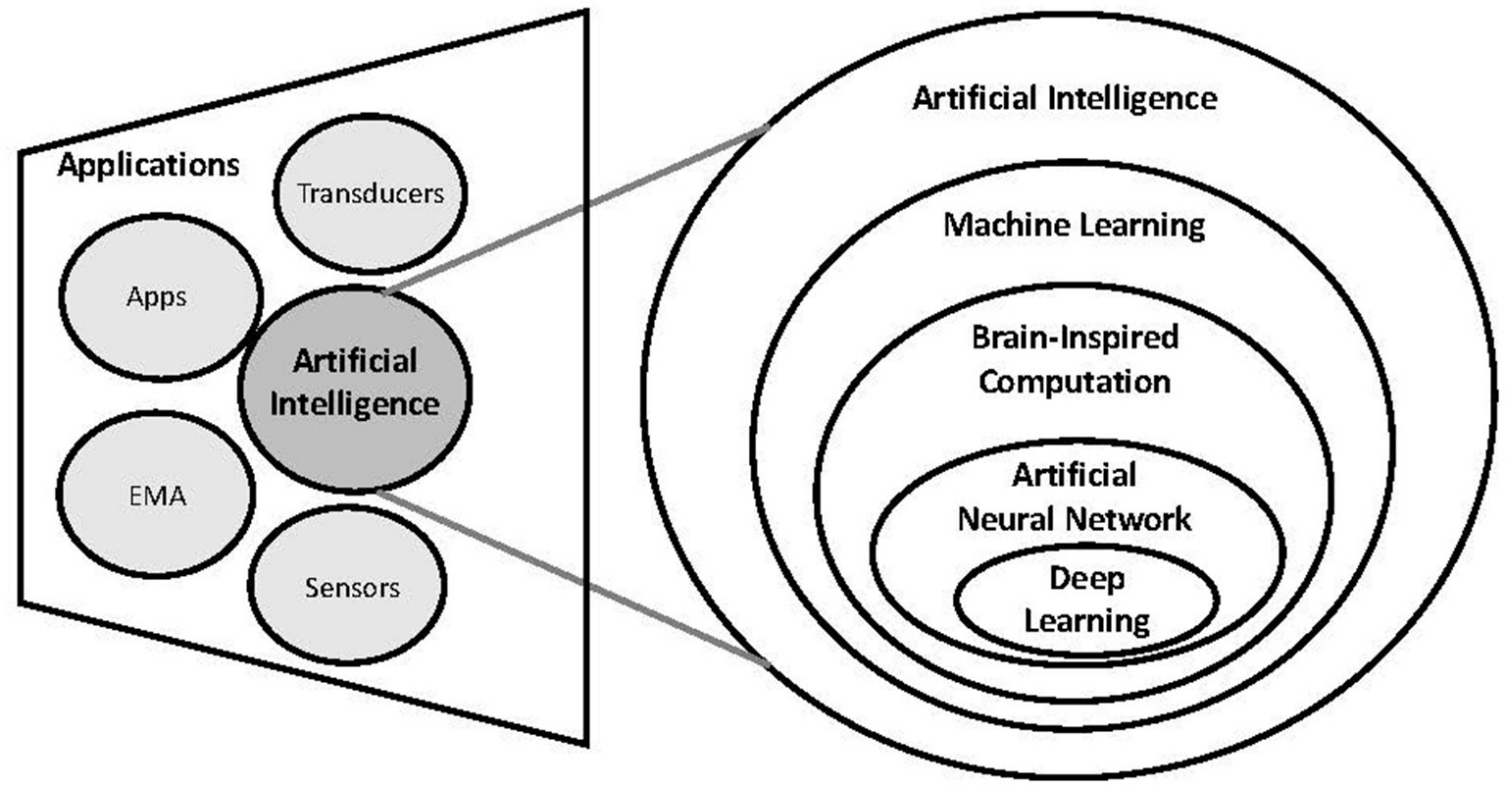
Analytical Artificial Intelligence methods and applications in Tinnitus.

The main AI methods that are being used in tinnitus research alongside current and potential AI-driven applications are described in subsequent sections (Figure 4).

#### Machine Learning in Tinnitus

ML is a field of study that applies the principles of computer and mathematical science and statistics to create computational models, which are used for future predictions (based on past data) and identifying patterns in data (159). The use of ML has increased in healthcare applications and has been applied to behavioral, EEG, functional Magnetic resonance imaging fMRI, and Functional near-infrared spectroscopy (fNIRS) data (163–165). The learning algorithms in ML can be divided into two main groups: supervised and unsupervised (166). Supervised methods are based on learning from labeled datasets to create a predictive statistical model based on mapping input data to an output decision (prognosis or diagnosis outcomes). In unsupervised learning, data is unlabeled and the algorithms learn from the input data to detect differences and categorized the data into different groups (166, 167).

ML algorithms solve several tasks including clustering, classification and prediction. Clustering is an approach in data mining, pattern recognition, and knowledge discovery (168). This aims to objectively organize data samples into homogeneous groups, where the data samples within a group are similar (168). Classification is used for decision making by categorizing the data samples into different classes (diagnosis labels), while prediction is to provide an early detection of an outcome (e.g., response to treatment) (169). In the tinnitus literature, the classification of brain data has often been done using different ML methods. One of the commonly used method is the Support Vector Machine (SVM) which is based on a supervised learning to detect the relationship between the data samples and their class label information (163, 170, 171). SVM learns from data to assign a hyperplane in an optimal position in the data space such that the samples are best separated with respect to their classes (159). Multilayer Perceptron (MLP) is a another ML approach which is a well-known architecture of ANN with supervised learning algorithms that can perform non-linear classification and prediction (160).

Although EEG is widely applied to tinnitus research, few ML methods have been developed to classify tinnitus patients from healthy people using EEG. Sun et al. (171) proposed a multi-view intact space learning method to distinguish EEG signals and classify the tinnitus patients from healthy people using a SVM classifier; with accuracy of 99%. Monaghan et al. (172) applied SVM techniques to classify (at the individual level) tinnitus from healthy people, based on their Auditory Brainstem Responses. Their findings showed the existence of objective features in neural activity generated by the inner ear and early auditory brain that vary between individuals with/without subjective tinnitus with quite high accuracy (80%) (172). This approach shows potential to be developed into a diagnostic tool.

In order to increase the classification accuracy, researchers have tried to use ML feature selection methods to extract the most important variables from EEG data. For example, Liu et al. (163) studied cortical/subcortical morphological neuroimaging biomarkers that may characterize idiopathic tinnitus using ML methods. They used a hybrid feature selection algorithm combining the F-score and sequential forward floating selection (SFFS). SFFS is a search algorithm that is used to reduce the dimensions of feature space to improve the computational efficiency; it removes irrelevant features or noise, without losing the informative patterns in the data. The results suggested a combination of 13 cortical/subcortical brain regions had the highest classification accuracy for effectively differentiating patients with tinnitus from healthy subjects (163). In addition to EEG data, Shoushtarian et al. (165) collected fNIRS data to differentiate tinnitus patients from control participants and to identify fNIRS features associated with tinnitus severity. The Naïve Bayes classifiers (a mathematical formula for determining probability of an outcome occurring, based on a previous outcomes) were used to classify patients with tinnitus from controls. An accuracy of 87.32% was obtained to distinguish patients with slight/ mild vs. moderate/ severe tinnitus (165). These findings show the feasibility of using fNIRS and ML to develop an objective measure of tinnitus that might enable clinicians to provide new treatment plans.

No treatment is currently able to eliminate the perception of tinnitus, but reducing its impact is possible (70). However, treatment is complicated by the large variability in tinnitus, and response to treatments, amongst sufferers (4). Research developing machine learning methods for early prediction of the effectiveness of tinnitus interventions based on the response of tinnitus individuals have been undertaken (165, 173, 174). For example, Schecklmann et al. (173), used a new cluster analysis based on the multimodal datasets including Positron-Emission Tomography (PET) and clinical variables, and extracted the most important predictor variables to improve accuracy. Their findings showed that clustering according to patients imaging data (PET data) is feasible and might provide a new approach for identifying tinnitus sub-types. Niemann et al. (174) developed a model to predict depression severity after outpatient therapy based on variables obtained before therapy among tinnitus sufferers. In this study, a decision tree classifier, which is a supervised ML model was used to split the data samples into different outcomes (tree leaves) by passing them through several decision nodes (tree nodes) and assigning them to proper branches in the tree (174). The results indicated an accuracy of 89% for detection of depression severity after treatment using data extracted from questionnaire answered before treatment. By incrementally reducing the number of features on predictive performance the set of predictive features (the number of questions) required may be minimized. Therefore, determinants of tinnitus-related distress provide valuable information about tinnitus categorization and desired therapy planning. Niemann et al. (175) also identified that gender-associated differences may facilitate a more detailed identification of symptom profiles. AI may heighten treatment response rates, and help to create access for vulnerable tinnitus populations that are potentially less visible in clinical settings (35). Niemann et al. (35) generated different regression models in the dataset and finally classified the samples with respect to various regressions.

Tinnitus patients' psychological symptom-based phenotypes comparison with tinnitus have been explored in a Gaussian mixture model (176). It was found that specific symptom profiles (e.g., anxiety) were significantly correlated with cochlear implant users' tinnitus characteristics. The Gaussian mixture model was found as a promising ML tool for identifying psychological symptom-based phenotypes.

#### Artificial Neural Networks in Tinnitus

To gain a mechanistic understanding of how tinnitus develops in the brain, we need to design a biologically plausible computational model that mimic both tinnitus formation and perception, then evaluate the preliminary models using brain and behavioral experiments (177). ANNs are computational models directly inspired by, and partially modeled on biological neural networks (160). They are capable of modeling and processing non-linear relationships between inputs and outputs in parallel (161). The brain is a highly interactive and deep learning network, but nearly all multivariate models employed in brain data analysis are linear and do not model interactions. Understanding the dynamic patterns of spatiotemporal brain data through traditional machine learning methods is limited because temporal features of the data manifest complex interactions that change dynamically over time. Therefore, it is crucial to develop new computational models that are capable of learning spatiotemporal interactions between multivariate data streams. Durai et al. (34) used a behavioral case series, alongside EEG and a brain-inspired artificial neural network model, to evaluate the effect of three masking sounds therapy on tinnitus and associated symptoms across 12 months. The method was able to predict sound therapy responders (93% accuracy) from non-responders (100% accuracy) using baseline EEG recordings. The authors further used ANN model to examine the effects of Acoustic Residual Inhibition on EEG function, as well as the predictive ability of the model (93%) (158). This approach may aid in the development of predictive models for treatment selection.

Despite advances in AI, relatively little is known on how best to incorporate it into health service delivery. Personalized modeling of tinnitus could enable classification/prediction of an individual patients profile (178). In contrast to global modeling (the conventional AI systems the create a computational model for the entire dataset), personalized modeling learns from the most relevant datasets for the individual (personalized subset of features and samples). It increases the model efficiency as a smaller block of data features can be selected and used for AI learning algorithms. Personalized AI models can be also used in tracking the effectiveness of a treatment over time and evaluate the treatment success at an individual level. This could, as an example, lead to application of an AI clinical decision tool to direct care toward higher probability of success treatments improving: tinnitus therapy outcomes, more efficient in in time and cost, in turn reducing burden to the wider community.

## Discussion

**Figure 5:**
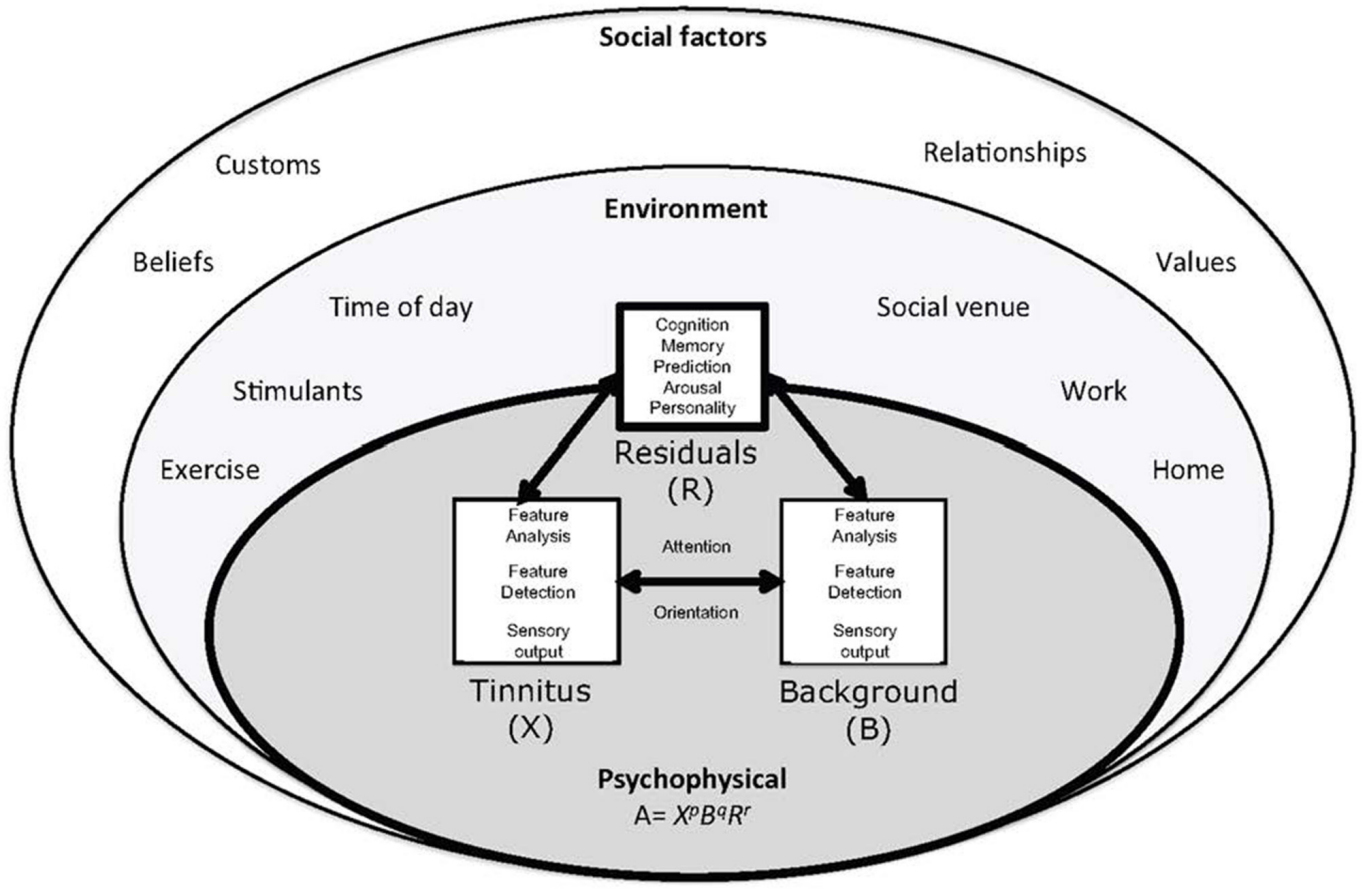
The ecological model of tinnitus. It consists of a psychophysical core described by adaptation level theory. In adaptation level theory tinnitus and background sound perception are under influence of individual psychology factors classified as “residuals.” These factors are influenced by the environment and social context. Helson (179) expressed this relationship mathematically: A = X^p^B^q^R^r^. The adaptation level (A) is the weighted product of: X, the intensity of tinnitus signal, *B*, intensity of background neural activity, and *R*, intensity of residual components (e.g., memory, arousal, and personality). The weighting coefficients *p, q*, and *r* determine the relative contributions of components to adaptation level. These factors are under the influence of environmental and psychosocial factors [(75) with permission of the authors].

The results of this review indicate that there are many digital technologies in use for tinnitus management, with an even greater number of technologies that demonstrate the potential to address the issue of tinnitus. A potential key to unlock success in tinnitus therapy is to address the heterogeneity of tinnitus and dispense targeted therapies (7). An individual's susceptibility to, and experience of, tinnitus is not divorced from the environment that surrounds them. Therefore, addressing multiple biopsychosocial factors (Figure 5) may be necessary to holistically treat tinnitus. Environment factors including circadian rhythms (87) and stress appear to interact with individual sensitivities (5, 75) to modulate tinnitus. A treatment method that could account for such modifiers would seem invaluable. The resolution in understanding an individual's tinnitus experience with and without treatment may be essential for very effective treatments, due to tinnitus heterogeneity. Physiological predictors for treatment effectiveness, and potentially adjustments for environment, may lead to highly personalized real-time adjustments to the individual and their environment. Sensor technology coupled with EMA and AI offers the promise of real-time delivery of personalized therapies.

### State-Of-the-Art Review Strengths and Weaknesses

This “state-of-the-art” review was undertaken to answer the question: what digital technology could be applied to tinnitus therapy in the next 10 years? State-of-the-art reviews are a subtype of narrative review that focus on current recent knowledge and highlight how research may advance this further. All review types have strengths and weaknesses (36). By focussing on the last decade this review has captured developments in the rapidly developing field of digital technology, that are, or could reasonably, be applied to tinnitus. The authors are knowledgeable in fields of behavioral science, audiology, artificial intelligence and engineering so are familiar with the topic and have been able to identify and fill gaps in the literature search by referring to missed peer reviewed publications and by use of gray literature. Gray literature includes manufacturer publications and consumer electronics publications. This literature does not provide high quality evidence, but it is current, and addresses commercial questions not commonly discussed in scientific papers. This review covers a breadth of material that a systematic review would reject as not meeting apriori quality criteria. The value of expertise from the authors must be considered in light of risk for bias:

“*a subject expert may simply provide a particularly idiosyncratic and personal perspective on current and future priorities*” [(36), p. 102].

With this is mind further research using scoping or systematic review methods, perhaps with a narrower focus, are recommended.

### Future Digital Therapeutics for Tinnitus and Research Priorities

**Figure 6:**
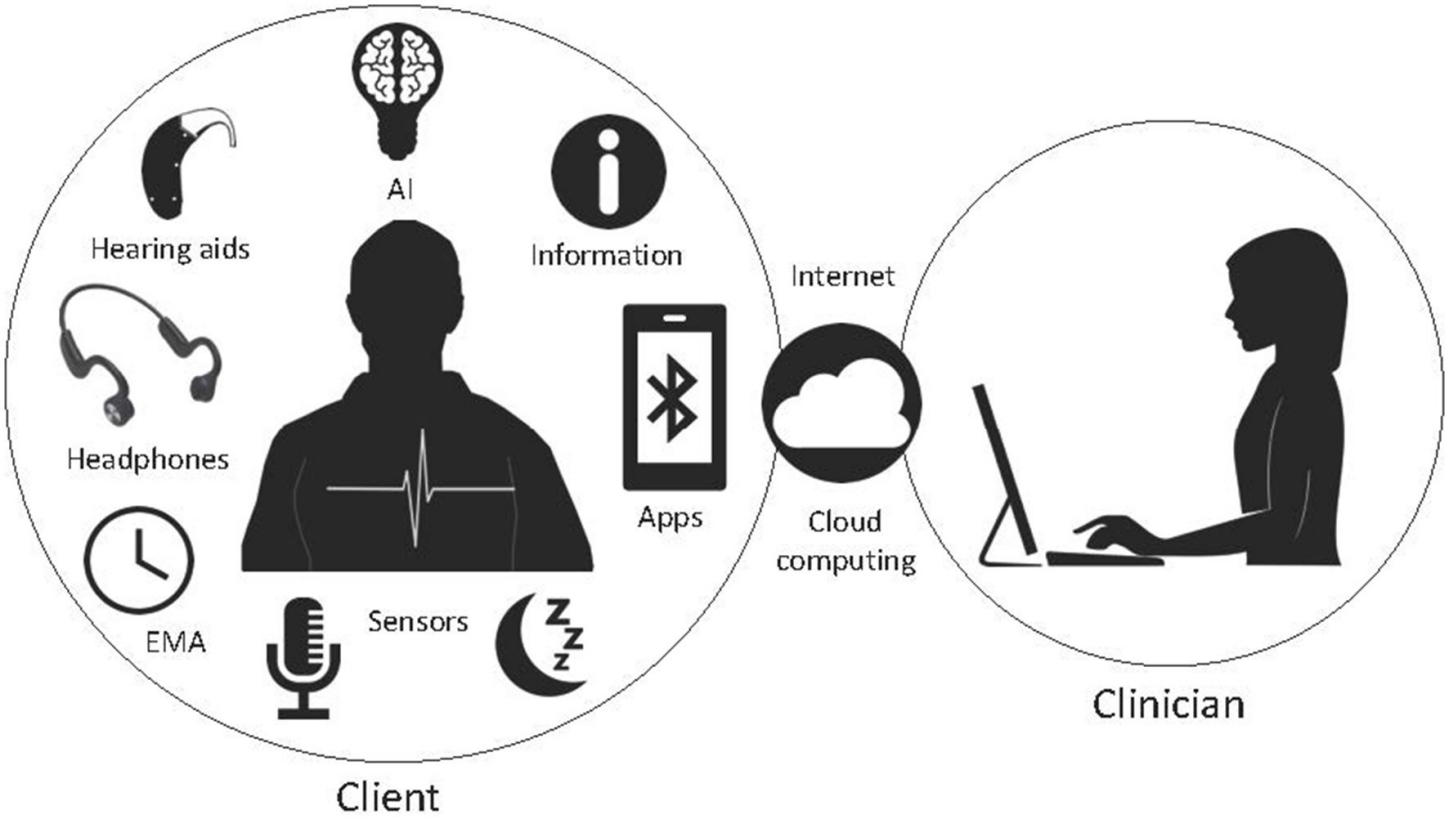
A potential future digital therapeutic system consisting of an app-based therapy that uses AI to configure transducers, counseling, EMA, biosensors and connect to clinicians via cloud computing.

Multifactorial treatments may be needed to address the diversity in tinnitus neurophysiology and patient goals. Recent development in smart mobile apps offers a large variety of functions that can be used for the clinical interventions and diagnosis in the chronic tinnitus. AI and machine learning tools could be used to learn from the trend of data and extract meaningful patterns for the purpose of precise prediction and classification of tinnitus, this information could inform counseling, hearing assistance and sound therapy. These concepts could be further developed toward making a smart therapy system for tinnitus, that may lend itself to use of AI decision tools and real-time treatment selection based on physiological markers. This will necessitate development of personalized AI models based on a group of individuals' data with similar characteristics. Our research group is working toward a Precision Sound Therapy™ that examines individual differences and treatment goals, and will employ AI to aid therapy selection (Figure 6).

The convergence of consumer and clinical devices is happening quickly in the hearing space; the release of the first Bose HA (180) the release of Jabra branded hearing aids by GN ReSound (181) and the purchase of Sennheiser by Sonova (64) being prime examples. Changes in the hearing aid space is likely to migrate to tinnitus therapy as well. The technical similarities between Hearables and HA are obvious (microphone, Bluetooth, signal processing, speaker/receiver) but the differences are still significant. Hearing access is used to promote Hearable devices, but their primary market is not, with a few exceptions, hearing impaired or tinnitus sufferers. Most Hearables are designed for entertainment and/or fitness tracking first while hearing impairment is a secondary concern, and potentially a marketing strategy (29). HAs are worn near continuously requiring low battery consumption and high comfort, need to be free of occlusion for voice quality and have to manage acoustic feedback, they also require different types of support than consumer electronics. Although universal devices may benefit from volume and mass production the majority of end-users do not have disabilities. This runs some risk that highly focused technology development undertaken by HA manufacturers could be compromised by the more generic solutions offered by consumer electronics companies. Importantly HAs are often accompanied by chronic medical conditions requiring clinical management, it is not clear how consumer-driven models will mitigate risk of undiagnosed pathology, especially when that consumer technology may mask the true problem, potentially delaying diagnosis. Debate as to the best model(s) for delivery of tinnitus management, self-help, self-directed and clinician led services also needs to occur for tinnitus.

This review has shown that clinicians and researchers in the tinnitus field do not lack imagination and innovation in their use of digital technology. However, many ideas appear to have been translated into commercial products before concepts are proven. To advance the field and develop effective digital therapeutics we suggest 6 key priorities for tinnitus technology research.

1. Tinnitus researchers should explore new and emerging technologies through appropriate proof-of-concept trials before the expense of randomized clinical trials and the lure of commercialization is entertained. Innovation is important, but not at the expense of evidence.
2. Physiological measures of tinnitus (or in their absence known associated measures) need to be included in trials as often as possible alongside behavioral measures so as to develop a reliable compendium of biomarkers for tinnitus therapy.
3. Wearable biosensors need to be applied with EMA to establish real-time patterns in tinnitus related physiology. The meaning and value of such measures need to be ascertained.
4. AI methods to adjust therapies to physiological-EMA measures need to be developed and tested to ascertain whether personalized tinnitus therapies can benefit from modifying response according to the patient's physiology and environment in real-time.
5. New health-delivery models should be developed with end-user communities. Data driven approaches need to ensure data privacy. Patient concerns regarding data use and data sovereignty need to be studied across cultures.
6. The role of the clinician in providing tinnitus digital therapies needs to researched from an efficacy, cost and consumer perspective. The CoVID-19 pandemic has illustrated the value of remote care and access to services outside clinic walls. The value proposition of new technology relative to established patterns of clinical care should be explored.

## Conclusions

The bourgeoning industry for digital tinnitus services is an exciting area but current opinion is that it should be used as an adjunct to, rather than a replacement of, clinical care. The uncertain mechanisms underpinning tinnitus present a challenge and many posited therapeutic approaches may not be successful. Some current therapies appear to be driven by technology innovation capability and theory rather than evidence. Due to the heterogeneity of tinnitus, response to various treatments differs between individuals. Holistic programs that offer multiple therapeutic facets such as sound therapy, multisensory stimulation, information, guided meditation, and counseling may address this heterogeneity. Personalized AI modeling based on biometric measures obtained through various sensor types, and assessments of individual psychology and lifestyles should result in the development of smart therapy platforms for tinnitus.

## Author Contributions

PS undertook the initial database search and initial consideration of title relevance to the study, he was primarily responsible for sections related to EMA and apps. ZD and MD reviewed articles related to AI. KS provided the review of stress and tinnitus. AB was responsible for the AR/VR sections. RB reviewed the technical accuracy of the manuscript. GS was primarily responsible for the introduction, reviews of hearing aids, hearables, cochlear implants, internet-based therapies, perceptual training, dedicated sound therapy devices, multimodal therapies, sensors, and the discussion, had overall oversight, and editorial responsibility for the review. All authors contributed to the synthesis of concepts and editing of the manuscript.

## Conflict of Interest

GS has commercial interests in tinnitus therapy, he is a director of Tinnitus Tunes a subscription based tinnitus therapy website and has received research funding from Hearing Aid manufacturers. The remaining authors declare that the research was conducted in the absence of any commercial or financial relationships that could be construed as a potential conflict of interest.

## Publisher's Note

All claims expressed in this article are solely those of the authors and do not necessarily represent those of their affiliated organizations, or those of the publisher, the editors and the reviewers. Any product that may be evaluated in this article, or claim that may be made by its manufacturer, is not guaranteed or endorsed by the publisher.
